# Resistance of Meningococcus to Intraspinal Serum Treatment

**Published:** 1918-11

**Authors:** 


**Resistance of Meningococcus to Intraspinal Serum Treatment.** By MARCEL BLOCH and PIERRE HEBERT. *Presse Médicale*, July 4th, 1918.

Very good results are obtained in the treatment of meningitis by early intraspinal injections with strong doses of serum, according to the principles of Netter, Dopter and others.

Nevertheless, certain forms of the disease are not amenable to the treatment and, at the present time, death caused by cerebro-spinal meningitis is by no means rare. The writers attribute this failure to three different causes.

1. Tardy intervention in cases which are serious from the start.

2. Resistance of the organisms — primitive or acquired — to the action of the serum.

3. Formation of meningeal septums isolating sections of the sub-arachnoid spaces, thus depriving those sections of the curative action of the serum.

The last cause has been most often referred to in the fatal cases recently published.

These meningeal septums have been identified by the writers on a level with the rachidian sack, or that of the encephalon, or at the base of the ventriculo sub-arachnoid spaces. The stoppage leads to pyocephalia.

In a fatal case the authors obtained a culture extremely fertile in meningococci in the ventricules, whereas spinal fluid had become sterile, providing the only absolute proof of the ventriculo sub-arachnoid stoppage.

This, however, is not general, since in other fatal cases, though false membranes had developed on the convolutions, the circulation of the spinal fluid had not been stopped, and it appears that other causes, besides these rachidian septums, must account for the resistance of the infection to spinal serotherapy.

The extraordinary evolution of two cases, one of which was fatal, seems to provide a new theory on the causes of resistance of meningococcic meningitis to the usual treatment. In both cases the germ disappeared; the spinal fluid was perfectly clear; but the meninges became suddénly reinfected and there was a distinctive relapse, so abrupt that one might think there was a rupture of an abscess in the subarachnoid spaces, which had been growing close to the meningeal bulb while the latter was becoming sterilized.

The circulation of the organism in the blood is easily proved. Clinically speaking, purpura, as M. Netter has demonstrated, is a proof of microbian embolism.

From these and other observations, the authors conclude that " during the septicemic state without meningitis, the meningococcus must have a seat in contact with the meninges ", which become irritated as the albumino-cytologic reaction of the fluid shows, without, however, being infected as the sterility and limpidity of the fluid also shows.

We must emphatically insist on admitting that in septicemias without meningitis we find " local centers " of the germ, as the very fact of these intermittent discharges invading the whole circulation testifies. We must also point out that these " local centers " are not in communication with the sub-arachnoid spaces, but in contact with them.

The writers remark that no lesions whatever are revealed on the nervous substance when cerebro-spinal autopsy takes place. The lesions are outside the pia-mater, and yet inside the pia-mater there are infiltrations of the perivascular sheaths. These, they think, may possibly be the center of infection.

It is, therefore, imperative to have a therapy adjuvant to the spinal serotherapy which is shown to be insufficient. The writers propose serum injections in the whole circulation, such being also the opinion of M. Nicolle.

Before the identification of the organism, the first injections should be with a mixture of serum A and B, or a polyvalent serum like M. Netter. Afterward the serum corresponding to the identified germ will be used.

